# The Double-Edged Sword: Unraveling the Dual Outcomes of Workplace Humor on the Social Identity of Employees

**DOI:** 10.5334/irsp.935

**Published:** 2024-10-08

**Authors:** Sana Mumtaz

**Affiliations:** 1 Master of Management, University of Niagara Falls, Canada

**Keywords:** deviant behavior, expressed rudeness, leader identity, social identity theory, surface acting, voice behavior, workplace humor

## Abstract

Building on the social identity theory, this conceptual article proposes a process model to understand the linkage between workplace humor and the social identity change experiences of employees. Further, it identifies the underlying mechanisms and conditions which impact the positive and negative identity changes during this process. Based on the proposed model, it is suggested that exposure to negative workplace humor is likely to lead to employee surface acting particularly when the need for social affiliation is high among individuals. On the one hand, identity synergy would facilitate positive emotions and psychological safety and is likely to support improved voice behavior in employees. On the other hand, perceptions of identity conflict would trigger negative emotions and lead to emotional exhaustion and expressed rudeness at the workplace; such individuals would engage in deviant workplace behaviors because of persistent negative experiences. Overall, the proposed conceptual model proposes a thorough relational process model unveiling socio-psychological outcomes of negative workplace humor and needs to be tested in multiple contexts to unveil the role of novel conditional factors impacting internalized change experiences of employees.

## Introduction

The critical role of employee emotions and their outcomes have remained a major area of discussion for researchers within the domain of sociology, psychology and organizational behavior (De Longis at al. 2022; Feinberg, Ford & Flynn 2020; Gkinko & Elbanna 2022; Svensson & Pesamaa 2018). In this regard, a considerable body of literature suggests how negative emotions lead to destructive impacts on the work performance of employees (Dahri et al. 2023; Lennard, Scott & Johnson 2019; Reynolds-Kueny & Shoss 2021) and positive emotions facilitates them in enhancing their performance (e.g., Lennard et al. 2019; Paakkanen, Martela & Pessi 2021). While the existing literature has explicitly identified constructive and destructive outcomes of positive and negative emotional experiences respectively, limited understanding exists regarding the linkage of emotional expressions with the long-term non-work change experiences of employees (e.g., Mumtaz 2019; Thompson et al. 2020). In addition, a lack of understanding exists regarding the cognitive and emotional processes of employees, and how those mechanisms lead to positive and negative changes in the social psychology of employees over time.

Overall, a sufficient body of literature has illustrated the crucial role of workplace humor and how it impacts the relationship development and emotional expressions of employees (e.g., Brender-Ilan & Reizer 2021; Pham & Bartels 2021). Thus, this research has focused on the antecedent role of workplace humor as it is a multifaceted complex instrument that leads to several positive or negative outcomes for employees (Evans & Steptoe-Warren 2018; Kong, Cooper & Sosik 2019). Further, humor is a major and frequently used socialization tool that leads to the development of an understanding among employees and leads to their eventual adjustment and learning in a work environment (Taylor, Simpson & Hardy 2022). Despite the dominant role of workplace humor in the development of new relationships in organizations and the presence of adequate literature on various types and outcomes of workplace humor, the existing literature has primarily linked humor with positive work-related outcomes (Oosthuizen 2021). However, there is a lack of understanding regarding the underlying mechanisms through which workplace humor leads to positive and negative changes in the social psychology of individuals.

In this regard, the social identity theory offers an in-depth socio-psychological perspective for understanding how and under what conditions interpersonal interactions lead to long-term and individualized employee changes (Tajfel 1982). This theory posits that when individuals interact with new groups, they engage in ‘self-categorization’ and evaluate the characteristics of new groups, which facilitate them in constituting positive or negative social identity (Jackson et al. 1996). A positive social identity assists in developing new social relationships, acceptance of internal changes, and positive self-esteem. In contrast, a negative social identity prohibits individuals from joining the membership of new groups and increases consciousness regarding protecting their identities (Mumtaz & Nadeem 2020). Overall, persistent exposure to negative and conflicting situations might trigger individuals to engage in counterproductive behaviors to protect their internal identities. Some of the existing literature has integrated the social identity theory to illustrate how exposure to workplace humor hinders or facilitates employees in experiencing long-term changes in their social psychology and behavioral responses over time (e.g., Taylor et al. 2022; Yang & Yang 2023).

Based on the comprehensive and multifaceted nature of the social identity perspective, this research has developed a linkage between workplace humor and the social identity change experiences of employees. To develop a comprehensive model, the framework has simultaneously embedded the positive and negative dimensions of identity change experiences to identify conditions under which exposure to workplace humor leads to constructive or destructive changes in employees. To provide a holistic perspective on outcomes of positive emotions, the crucial role of psychological safety, identity synergy and the need for social affiliation have been incorporated to illustrate a positive social identity change in the voice behavior of employees. Contrary to the above, the role of emotional exhaustion, expressed rudeness and identity conflict have been embedded in a model to identify how employees would engage in deviant behavior over time.

The contributions of this conceptual article are threefold. First, it develops a unique process model for understanding the linkage between workplace humor and social identity changes in employees. While most of the existing literature has linked emotional expressions with the work experiences of employees, this research goes beyond current discussions and identifies how exposure to workplace humor leads to non-work changes in the social psychology of individuals. Second, unlike the focus of most of the existing organizational behavior literature on resource and personality-based perspectives, this research has integrated a comprehensive process model, social identity theory, to simultaneously focus on the positive and negative outcomes of workplace humor on the social identity of employees. Third, the proposed model in this research has developed a process model by identifying the intervening role of several crucial factors such as surface acting, emotional experiences, psychological safety and emotional exhaustion and expressed rudeness to depict the step-wise change experiences of employees. Further, it has unveiled the conditional role of factors such as the need for social affiliation, identity conflict, identity synergy and leader identity to depict specific conditions under which employees experience favorable or unfavorable internalized changes over time.

## Literature Review and Framework Development

**Figure 1:**
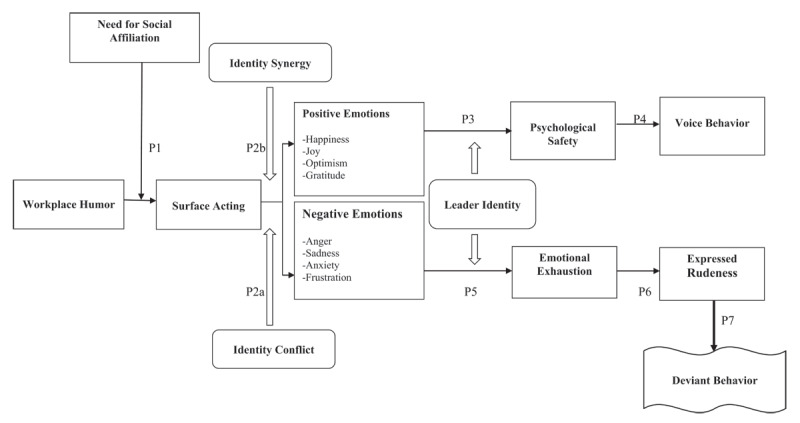
A Process Model Linking Workplace Humor with Positive and Negative Social Identity of Employees.

This section presents a review of the key variables and develops a process model linking workplace humor with positive and negative internalized change experiences of employees through the lens of social identity theory. In addition, it identifies the essential role of intervening and conditional factors in the above relationship; the proposed framework is presented in Figure 1.

### Workplace Humor and Surface Acting

The concept of ‘humor’ refers to the manifestation of content amusingly and entertainingly through the use of verbal and non-verbal communication (Martineau 1972). Further, it helps individuals look at the phenomenon from a fun perspective (Romero & Cruthirds 2006). Despite the importance of humor in different spheres of life, humor in workplaces has become increasingly popular because employees face intense pressure and complex challenges in a work environment (Brender-Ilan & Reizer 2021; Evans & Steptoe-Warren 2018). Overall, humor tends to facilitate individuals in reducing their stress and normalizing their behavior towards colleagues through their occasional participation in amusing discussions. Most of the existing literature links workplace humor with positive emotions (Romero & Cruthirds 2006) and performance outcomes such as creativity (Lang & Lee 2010), while a limited body of literature suggests how negative workplace humor leads to adverse outcomes in organizations (e.g., Chen & Ayoun 2019).

In this regard, the social identity theory guides that when individuals initiate discussions with members of new groups, they tend to exhibit proactiveness and conformity to develop positive intergroup ties (Abrams & Hogg 1990). Based on the above guidance, the organizational behavior literature also suggests that when employees work in organizations, they engage in interpersonal interactions with coworkers and supervisors for information-seeking and to enhance their learning about the existing work and non-work practices (Kammeyer-Mueller et al. 2013). In doing so, employees consciously regulate their emotional displays and express positive behaviors towards their colleagues to maintain positive social interactions (Hu & Shi 2015). It is expected that when employees maintain regular interactions with coworkers and supervisors, they become a part of informal discussions. Although positive responses to workplace humor facilitate employees in quickly learning the informal norms and culture of an organization (Mak 2008), it is important to recognize that inappropriate and negative workplace humor, such as aggressive, offensive or sarcastic jokes, lead to negative emotional responses among individuals (Davidhizar & Shearer 1997). Based on the pressures of managing adjustment in the workplace, it is proposed that employees are likely to hide their original responses after coming across such situations and prefer engaging in surface acting (e.g., through fake laughter) to demonstrate comfort in group settings. Surface acting refers to the exhibition of unreal behaviors when individuals experience a lack of compatibility between their inner feelings and displayed emotions. Thus, people tend to disguise their true emotions and portray fake behaviors (Grandey 2003).

Importantly, it is crucial to understand the conditional role of the need for social affiliation in the relationship between workplace humor and surface acting by employees. The need for social affiliation refers to the desire to maintain close relationships with others (Bloemer, Odekerken-Schroder & Kestens 2003). Overall, it encompasses the need for companionship, social interaction, and belongingness. People with a high need for social affiliation seek out and value social connections, enjoy engaging in social activities, and often prioritize building and nurturing relationships in their personal and professional lives (Cheng et al. 2023; Volmer et al. 2021). Thus, it is expected that a high need for social affiliation would enhance the vulnerability of employees in social groups and tend to encourage surface-acting behaviors for maintaining their social statuses in work groups. Based on the above discussion, Proposition 1 is presented.

**Proposition 1:** Negative workplace humor leads to surface acting by employees particularly when they have a high need for social affiliation.

### Outcomes of Surface Acting on Emotions of Employees

A large body of existing organizational behavior has developed a linkage between surface acting and negative outcomes such as emotional exhaustion, increased stress and weak performance (e.g., Grandey 2003; Krannitz at al. 2015). Most of this literature has integrated Fredrickson’s (2001) conservation of resource model to depict how the display of fake expressions leads to weak energy and quick depletion of psychological resources among employees. However, there is a weak theoretical understanding regarding circumstances in which surface acting leads to various negative emotions among employees. To develop this linkage, the social identity theory has been used, which suggests that individuals try to engage in proactive communication with new groups. However, the lack of compatibility and conflicts leads to negative social identity changes and increases gaps between them (Mummendey et al. 1999). In line with the above, some organizational behavior also hints that surface acting triggers negative emotions such as anger and anxiety among employees and reduces interactions between two groups (Kumar, van Kleef & Higgins 2019). Hence, it is suggested that persistent surface acting leads to negative emotions such as anger, sadness, anxiety and frustration in employees.

Further, the personal identity of individuals plays a major role in understanding the above relationship (Stets & Burke 2000), as individuals who experience identity conflict are likely to become more vulnerable and face stronger negative emotions. Identity conflict refers to a situation when there is a lack of incongruence between the personal and social identities of individuals (Leong & Ward 2000). Moreover, individuals feel psychologically stressed when their internal values and principles clash with their external experiences (Hirsh & Kang 2016). Thus, it is argued that employees who experience a lack of alignment between their internal values and that of colleagues’ identities are likely to experience strong negative emotions after engaging in surface acting. Based on the above discussion, Proposition 2a has been developed.

**Proposition 2a:** Surface acting leads to negative emotions (anger, sadness, anxiety, frustration) among employees when they experience identity conflict while interacting in work groups.

There has been a consensus in the existing literature regarding the negative nature of surface acting and how it leads to negative emotional experiences for employees (e.g., Grandey 2003; Zhang et al. 2018). However, limited literature hints at how surface acting leads to positive changes among individuals (e.g., Lennard, Scott & Johnson 2019). It is argued that a unique perspective of this relationship may be unveiled through the social identity theory, which suggests that frequent interactions facilitate individuals in deepening an understanding of others’ perspectives and lead to the eventual acceptance of group memberships (Tajfel 1982). It is crucial to understand that personality traits and learned characteristics also influence the nature of the relationship between surface acting and positive emotional experiences, which may be explained through the concept of identity synergy. Identity synergy refers to the development of alignment between the personal and social identity of an individual and facilitates a person in configuring behaviors following the external environment (Heger & Gaertner 2018). Moreover, identity synergy psychologically allows individuals to maintain multiple identities at one point in time (Fombelle et al. 2012). Thus, it is suggested that employees who experience the development of identity synergy after interaction with their work colleagues are more likely to feel comfortable in their new relationships and hence experience positive emotions such as happiness, joy, optimism and gratitude.

Using the social identity theory, it is suggested that employees initially engage in surface acting and display positive fake emotions when negative humor is experienced at workplaces. However, daily interactions and frequent discussions facilitate employees in aligning their mindsets and beliefs with other colleagues through developing a deeper understanding of beliefs and mindsets of other employees (Morrison & Cooper-Thomas 2016; Mumtaz & Nadeem 2022). Overall, deeper understanding and frequent interactions would encourage employees to adapt to work interactions and facilitate in experiencing positive emotions such as happiness, joy, optimism and gratitude. Based on the above discussion, Proposition 2b has been developed.

**Proposition 2b:** Surface acting leads to positive emotions (happiness, joy, optimism, gratitude) among employees when they experience identity synergy while interacting in work groups.

### Outcomes of Positive Emotions on the Social Identity of Employees

When employees experience positive emotions at the workplace, it leads to several favorable outcomes in organizations, such as improvement in work performance and job enrichment (Staw, Sutton & Pelled 1994). Overall, the organizational behavior literature has developed an adequate linkage between positive emotions and conducive performance outcomes. However, it is essential to understand how positive emotions lead to individualized changes in employees’ social identity with time. Some literature on organizational behavior suggests that positive emotions such as happiness and appreciation develop trust among team members. When employees experience and express positive emotions, it helps create a trusting environment where individuals feel psychologically safe (Lee 2021). Similarly, positive emotional experiences promote a constructive approach to giving and receiving feedback, which enhances psychological safety among employees by ensuring that employees feel comfortable expressing their thoughts and opinions (Frazier et al. 2017). Thus, it is argued that positive emotions are likely to enhance psychological safety by strengthening employees’ identification with their workgroups.

In addition, the role of leader identity plays an integral role in the above relationship. A strong leader identity is characterized by a clear, authentic, and consistent self-concept that aligns with effective leadership qualities and inspires trust, respect, and engagement from followers (Miscenko, Guenter & Day 2017). It is suggested that when leaders have a strong identity that aligns with supportive and inclusive behaviors, employees are more likely to feel valued and secure, enhancing their psychological safety. Conversely, a weak leader’s identity does not align with these positive attributes and employees might be less responsive to positive emotions, potentially weakening the connection between positive emotions and their psychological safety (Wang & Shi 2021). Thus, the alignment between a leader’s strong identity and positive emotional expressions can significantly influence how effectively positive emotions contribute to creating a psychologically safe work environment. Based on the above discussion, Proposition 3 is developed.

**Proposition 3:** Positive emotions (happiness, joy, optimism, gratitude) lead to psychological safety among employees particularly when leader identity is strong in organizations.

The social identity theory asserts that the development of new groups facilitates individuals in understanding the perceptions and attitudes of other members (Ashforth & Mael 1989). As a result of embracing new memberships, individuals adopt changes in behaviors with time (Abrams & Hogg 1990). Such changes may be experienced by developing new social groups, referred to as in-group ties, development of emotional attachment, commonly known as in-group affect, or through the conscious awareness of new memberships, referred to as centrality (Cameron 2004). Some organizational behavior literature guides how psychological safety experiences facilitate employees in interpersonal engagement and enhance internal motivation among employees (Huang, Shen & Yuan 2022). Further, improved psychological well-being of employees helps them in accepting changes in their beliefs and attitudes as it encourages employees to spend quality time with their colleagues and supervisors and leads to the improved voice behavior of employees (Mumtaz & Aqif 2023). Voice behavior refers to employees’ proactive efforts to speak up, offer constructive suggestions, or express concerns about work-related issues, aiming to improve organizational processes or address problems (Nikolaou, Vakola & Bourantas 2008). Further, it involves the willingness to share ideas and feedback, contributing to positive change and enhancing workplace effectiveness (Morrison 2011).

Using social identity theory, it is argued that psychological safety leads to positive social identity changes in employees by fostering a strong sense of belonging and trust within a team, encouraging employees to share their ideas and concerns without fear of negative consequences (Liu et al. 2016). When employees feel securely integrated into their workgroup, they are more likely to engage in voice behavior, believing their contributions will be valued and accepted. This positive social identity reinforces their willingness to participate openly, ultimately driving innovation and improvements within the organization (Lee, Loretta Kim & Yun 2023). Based on the above discussion, Proposition 4 has been developed.

**Proposition 4:** Psychological safety improves the voice behavior of employees.

### Outcomes of Negative Emotions on the Social Identity of Employees

Negative emotional states have been persistently linked with the internal psychology of individuals in a large body of literature (e.g., Xu, Luo & Hsu 2020; Zheng & Montargot 2022). This literature suggests that when individuals persistently experience negative emotions (such as anger, sadness, anxiety and frustration) in the workplace, the impact of such bitter experiences may lead to attitudinal changes in them (Kiefer 2005). This is congruent with the idea of the social identity theory, which focuses on internal psychological mechanisms and suggests that individuals experience unconscious changes in their behaviors due to interactions with new social groups (Cameron 2004). Some literature guides that negative emotions can lead to emotional exhaustion in employees when these emotions disrupt their sense of belonging and alignment with their workgroup (Thompson et al. 2020). When employees experience negative emotions, it can erode their identification with the team, leading to a diminished sense of support and cohesion. This breakdown in group identity can result in increased stress and emotional fatigue as employees struggle to reconcile their negative feelings with their role within the team. Thus, negative emotions undermine the supportive group dynamics necessary for preventing emotional exhaustion (David et al. 2020).

In addition, leader’s identity is likely to moderates the relationship between negative emotions and emotional exhaustion. When leaders lack a strong and defined identity, they may struggle to effectively address or mitigate the negative emotions employees experience, leading to a weaker sense of group cohesion and belonging. Overall, this lack of effective leadership can exacerbate employees’ feelings of frustration, intensifying their emotional exhaustion with time (Liu et al. 2020). Consequently, weak leader identity can amplify the negative impact of emotions on emotional exhaustion, as employees may feel less supported and more vulnerable. Based on the above guidance, Proposition 5 has been developed.

**Proposition 5:** Negative emotions (anger, sadness, anxiety, frustration) lead to emotional exhaustion among employees particularly when leader identity is low.

There has been a consensus in the organizational behavior literature that emotional exhaustion depletes employees’ psychological resources and diminishes their ability to engage positively with colleagues to effectively manage interpersonal relationships (e.g., Parray, Islam & Shah 2023; Thompson et al. 2020). Overall, emotional exhaustion can lead to increased expressed rudeness among employees when their diminished sense of belonging and group cohesion erodes their capacity for empathy and patience. As employees experience emotional exhaustion, their ability to manage stress and maintain positive interpersonal interactions weakens, resulting in more frequent displays of rudeness (Welbourne, Miranda & Gangadharan 2020). Expressed rudeness refers to unjustified insensitive and disrespectful behavior toward other colleagues (Porath & Erez 2007). Further, although individuals don’t intend to harm other employees by exhibiting impolite behavior, it generally reflects a violation of social norms and carries several adverse implications (Blanz et al. 1998). Using the social identity theory, it is argued that reduced emotional energy and frustration can intensify conflicts and decrease tolerance for others, undermining the supportive group dynamics necessary for courteous behavior (Adamovic 2022). Consequently, the strain on social identity due to emotional exhaustion can lead to a higher incidence of rudeness as employees struggle to cope with their reduced sense of belonging and support within the organization.

**Proposition 6:** Emotional exhaustion leads to expressed rudeness of employees.

The literature on organizational behavior has identified several negative outcomes of rudeness at work, such as weak performance and reduced well-being of employees (e.g., McCarthy 2016). However, based on the complex and multifaceted nature of this behavior, it is important to explore how expressed rudeness at the workplace psychologically impacts the mindset and attitude of individuals in the long run. However, limited research has examined how individuals experience changes in their internal psychological mechanism after displaying incivility at the workplace (e.g., Mumtaz & Aqif 2023). Most of the existing literature on the social identity theory focuses primarily on processes and mechanisms that lead to the development of new group memberships and positive social identity changes (e.g., Ashforth & Mael 1989; Jackson et al. 1996). However, recent literature has used the social identity theory to focus on the negative social identity of individuals as well (e.g., Mumtaz & Nadeem 2020) and suggests how individuals experience a negative change in their social identity due to a lack of alignment between their values and social identity of other groups.

The above research suggests how continuous exhibition of negative behaviors spread like a contagion to the internal mechanism of individuals and leads to deviant behavior of employees (Zahid & Nauman 2024). Deviant behavior refers to employees’ actions or conduct that violate organizational norms, rules, or expectations, such as unethical practices, insubordination, or disruptive behavior (Appelbaum, Iaconi & Matousek 2007). Such types of behavior can undermine workplace harmony, reduce productivity, and damage the organization’s culture and reputation. It is argued that when negative emotions and a weakened sense of group belonging lead to rule-breaking or disruptive actions, it may lower their adherence to organizational norms and increase the likelihood of engaging in deviant behaviors (Han, Kang & Park 2020). Rudeness, stemming from frustration or alienation, may manifest as a form of rebellion or resistance against perceived injustices or lack of support, further fueling deviant actions. Examples of employee deviant activities may include engaging in unauthorized use of company resources and exhibiting insubordinate behavior, such as openly defying management directives or policies. Such deviant behavior activities may disrupt workplace norms and can lead to decreased productivity and a toxic work environment (Robinson, Wang & Kiewitz 2014). Based on the above discussion, Proposition 7 has been developed.

**Proposition 7:** Expressed rudeness leads to deviant behavior of employees over time.

## Discussion

Integrating the social identity perspective, this research has proposed a conceptual model to develop a linkage between workplace humor and socio-psychological change experiences of employees. This section has elaborated some of the key theoretical contributions as well as offered specific directions for advancing theoretical knowledge in this field.

First, unlike the focus of most of the literature on understanding the work-based outcomes of humor, the conceptual framework in this research has unveiled the relationship between work humor and non-work change experiences. While workplace humor has been associated with positive relationships, it has been argued that workplace humor may lead to discomfort for employees and encourage them to engage in surface acting to protect their social positions. This conceptual model is especially relevant in the context of employees whose need for social affiliation is high, which makes them vulnerable in such circumstances. While a basic framework has been developed to propose the above relationships, future researchers are encouraged to test this framework in various contexts to understand relevance of these relationships across various cultures. In addition, humor is a relatively complex and multifaceted term, which is interpreted differently in different cultures, thus future researchers are encouraged to compare various types of humor, such as sarcastic and aggressive humor, to understand how exposure to various categories of humor leads to unique changes in employees. In addition, this research has embedded ‘need for social affiliation’ as a key factor in the model; however, future researchers are encouraged to embed other essential factors, for example, personality attributes to unveil varied responses of employees. Based on the emerging use of remote/hybrid work arrangements, researchers may explore how modern technologies facilitate or hinder change in workplace humor dynamics and feedback mechanisms. Also, researchers are recommended to explore the patterns and effectiveness of virtual communication in building and maintaining professional relationships compared to in-person interactions.

Second, while most of the existing literature has predominantly focused on identifying the positive work outcomes of workplace humor (e.g., Lennard et al. 2019; Paakkanen et al. 2021), the conceptual framework in this research has adopted an impartial approach to comprehensively cover the positive as well as negative side of the workplace humor through the social identity lens. Overall, the simultaneous focus of social identity theory on positive and negative social identity changes has facilitated in understanding how the two distinct groups accept or reject novel changes under unique conditions. Despite the simultaneous focus of the proposed model on both (positive and negative) aspects of social identity, ‘negative social identity changes’ have gained limited attention in the social identity literature, hence future researchers are suggested to theorize negative social identity changes to understand how pessimistic emotional experiences translate to negative behaviors with time. In addition, incorporating other theoretical models, including theory of identity threat and social comparison theory, might help researchers in deeply understanding the role of personal identities of individuals in the above process and how those identities prevent individuals from engaging in deep interpersonal interactions.

Finally, this research has developed internal socio-psychological mechanism which hasn’t been thoroughly investigated in the existing research. On the one hand, it is argued that negative emotions would lead to emotional exhaustion and expressed rudeness of employees, and employees are likely to experience negative social identity changes by engaging in deviant behavior, particularly when leader identity is weak. On the other hand, positive emotional expressions would lead to psychological safety and enhanced voice behavior of employees over time. Overall, this model has focused on identifying positive and negative behavioral and attitudinal responses of employees. However, future researchers are recommended to particularly focus on unveiling negative social identity changes of employees. While a thorough process model has been developed through identifying the role of individual factors in the relational model, future researchers are recommended to identify and integrate the role of team and organizational level factors to develop further understanding about negative social identity changes of employees. In this regard, researchers are recommended to embed conditional factors such as ‘organizational culture’ and ‘team dynamics’ to unveil the complexity of negative change processes across various types of organizations. Further, negative behavioral social identity changes have been identified in this model (i.e., deviant behaviors); however, future researchers are suggested to focus on other negative social identity outcomes. Future researchers may unveil how persistent exposure to negative emotions leads to loneliness and psychological detachment of employees over time, for example. For this, researchers may analyze how modern technologies, such as social media and remote work platforms, contribute to or mitigate negative social identities among employees in a modern business environment.

### Practical Implications

The conceptual model in this research may facilitate organizations in effectively managing the work relationships between employees. Based on the proposed relationships in the framework, some practical implications are offered in this section. First, organizations are recommended to understand the positions of employees working at various organizational levels and how they face challenges of managing their socialization while working in a work environment. Based on the above, organizations are advised to facilitate employees in the socialization process and develop explicit policies regarding socialization mechanism in the work environment. In particular, based on the use of remote/hybrid work arrangements in the recent years, organizations are recommended to develop and communicate clear guidelines for acceptable professional humor in digital interactions. Also, they need to ensure how these guidelines/policies address tone, content, and context to maintain respect and inclusivity in a modern work environment. Importantly, human resource departments are recommended to provide channels for employees to give feedback on digital communication and humor. This can help managers understand the impact of humor and adjust modes of interactions as needed.

Second, workplace humor is helpful in developing a healthy relationship between employees; however, a lack of understanding regarding the sensitivity of this concept would lead to destructive impacts on the social identity of employees as suggested in this research. To improve this, understanding, supervisors are encouraged to model appropriate behavior and exemplify the behavior they expect from their teams. Also, they should encourage positive peer influence by promoting collaborative work and peer support. In addition, organizations are encouraged to organize frequent training for employees to familiarize them with the differences between positive and negative humor, and how it is crucial for employees not to violate someone’s personal space through their humor. Moreover, offering training sessions on digital communication skills, including how to use humor effectively in a hybrid/ remote environment would employees understand the nuances of humor in a digital setting. In addition, provision of trainings on empathy, self-regulation, and interpersonal skills would encourage emotional intelligence among employees and improve quality of interactions through improved coping mechanism.

Finally, this research has identified an essential role of emotional expressions and positive and negative behavioral responses of employees in a work environment. Based on the above, organizations are advised to remain cautious about observing employees’ attitudes at work. Overall, managers are recommended to create channels for open communication where employees feel comfortable sharing their feelings and experiences. This can help address concerns early and foster a supportive environment. Overall, fostering a culture of accountability by encouraging employees to take responsibility for their actions would promote transparency and honesty in all forms of digital communications. In addition, departmental managers are recommended to organize monthly one-on-one meetings with employees to understand if they are facing persistent stress or negative emotions in a digital work environment. Such regular meetings might help organizations in timely identifying the problems of employees. Thus, appropriate training or mentorship may be provided to save employees from becoming alienated in work groups. To facilitate employees experiencing negative social identity, offering resources such as access to mental health support, counseling, or wellness programs would facilitate employees manage their emotions and maintain well-being in the long run.
